# Triple Combinations of Histone Lysine Demethylase Inhibitors with PARP1 Inhibitor–Olaparib and Cisplatin Lead to Enhanced Cytotoxic Effects in Head and Neck Cancer Cells

**DOI:** 10.3390/biomedicines12061359

**Published:** 2024-06-19

**Authors:** Dawid Dorna, Robert Kleszcz, Jarosław Paluszczak

**Affiliations:** 1 Poznan University of Medical Sciences, Doctoral School, Department of Pharmaceutical Biochemistry, 60-806 Poznan, Poland; dawid.dorna97@gmail.com; 2 Poznan University of Medical Sciences, Department of Pharmaceutical Biochemistry, 60-806 Poznan, Poland; kleszcz@ump.edu.pl

**Keywords:** head and neck cancer, histone lysine demethylase, ML324, CPI-455, GSK-J4, JIB-04, olaparib, cisplatin, DNA damage, chemosensitization

## Abstract

PARP inhibitors are used to treat cancers with a deficient homologous recombination (HR) DNA repair pathway. Interestingly, recent studies revealed that HR repair could be pharmacologically impaired by the inhibition of histone lysine demethylases (KDM). Thus, we investigated whether KDM inhibitors could sensitize head and neck cancer cells, which are usually HR proficient, to PARP inhibition or cisplatin. Therefore, we explored the effects of double combinations of KDM4–6 inhibitors (ML324, CPI-455, GSK-J4, and JIB-04) with olaparib or cisplatin, or their triple combinations with both drugs, on the level of DNA damage and apoptosis. FaDu and SCC-040 cells were treated with individual compounds and their combinations, and cell viability, apoptosis, DNA damage, and gene expression were assessed using the resazurin assay, Annexin V staining, H2A.X activation, and qPCR, respectively. Combinations of KDM inhibitors with cisplatin enhanced cytotoxic effects, unlike combinations with olaparib. Triple combinations of KDM inhibitors with cisplatin and olaparib exhibited the best cytotoxic activity, which was associated with DNA damage accumulation and altered expression of genes associated with apoptosis induction and cell cycle arrest. In conclusion, triple combinations of KDM inhibitors (especially GSK-J4 and JIB-04) with cisplatin and olaparib represent a promising strategy for head and neck cancer treatment.

## 1. Introduction

Head and neck squamous cell carcinomas (HNSCCs) are the most prevalent malignancies that develop in the head and neck region. About 900,000 new cases and more than 450,000 deaths in 2020 place HNSCCs as the eighth most common cancer worldwide [1]. Significant progress in oncology has resulted in the introduction of molecularly targeted therapies and immune response modulators that significantly prolong patients’ survival compared to conventional treatments for many types of cancer. However, in the case of HNSCCs, very few modern drugs have been approved, and their efficacy has turned out to be limited [2]. Additionally, surgical resection of lesions, which is the basis of therapy, often results in permanent disfigurement due to the location of malignancies. Thus, it is important to look for more efficient and less debilitating therapeutic strategies for patients with HNSCCs.

Cisplatin and other chemotherapeutic drugs (5-fluorouracil, paclitaxel) remain an important form of treatment in patients with locoregionally advanced and metastatic HNSCC; however, the appearance of resistance due to various mechanisms limits the efficacy of chemotherapy [3,4]. Cisplatin should block replication and lead to cell death by causing the formation of DNA adducts at a level that is beyond the capacity for repair; however, cancer cells may upregulate the DNA damage response and evade apoptosis [5]. In general, cisplatin-induced damage may be repaired by the nucleotide excision pathway (NER). On the other hand, unrepaired lesions can lead to the formation of double-strand breaks, which can be repaired by the homologous recombination (HR) pathway [3,5,6]. Indeed, cisplatin resistance and poor prognosis in HNSCC patients have been associated with increased capacity in NER and HR repair pathways [3]. 

Moreover, it has been suggested that HNSCC patients showing the BRCAness phenotype, i.e., bearing mutations in the genes of the HR pathway, could possibly benefit from treatment with poly(ADP-ribose)-polymerase 1 (PARP-1) inhibitors [7]. PARP-1 was initially associated with the base excision repair (BER) pathway; however, more recent findings point to its role in all major DNA repair pathways [8]. Importantly, PARP-1 inhibitors (e.g., olaparib) exert so-called synthetic lethality in patients carrying mutations in HR pathway genes (the BRCAness phenotype), and they are used in the treatment of breast and ovarian cancer patients who carry *BRCA1/2* mutations [9]. Thus, it was suggested that olaparib could lead to cisplatin chemosensitization in HNSCC patients with mutated HR pathway genes [7]. However, the therapeutic effects of PARP-1 inhibitors in HNSCC treatment remain largely unexplored. Additionally, there is the question of the utility of using PARP-1 inhibitors in treating the majority of HNSCC patients who do not show the BRCAness phenotype [10].

Histone lysine demethylases (KDMs) are a group of enzymes catalyzing the removal of methyl groups from lysine residues in histones, and they play important roles in the regulation of chromatin organization and transcription. KDMs are aberrantly expressed in HNSCC and contribute to the neoplastic phenotype [11,12]. Importantly, several KDMs play key roles in DNA damage response signaling [13,14], and they can be regulated by PARP [15]. Indeed, a recent study has shown that the inhibition of KDM4-6 attenuated the HR-mediated repair of cisplatin-induced DNA damage by downregulating CtIP and led to cisplatin sensitization in lung cancer cells [16]. Thus, similarly to other epigenetic modulators [17], KMD4-6 inhibitors could pharmacologically mimic BRCAness and lead to the disruption of the HR pathway. Therefore, there is a possibility that the joint application of PARP-1 and KDM4-6 inhibitors could have a synergistic cytotoxic effect by simultaneously disrupting HR and other mechanisms of DNA damage repair and causing genomic instability. Interestingly, synthetic lethality between *KDM6A* loss and PARP inhibition was suggested in leukemic cells [18]. Additionally, the simultaneous pharmacological disruption of multiple DNA repair pathways by the proposed combination of compounds is likely to increase cellular susceptibility to DNA-damaging agents like cisplatin. This offers the possibility of tackling chemoresistance. This has prompted our investigation into whether KDM inhibitors could synergize with PARP inhibitors like olaparib in HNSCC cancer cells, in which *BRCA* mutations are infrequent. 

Therefore, the aim of this study was to assess whether combinations of selective inhibitors of KDM4 (ML324), KDM5 (CPI-455), KDM6 (GSK-J4), or a non-selective inhibitor of KDM4-6 (JIB-04) with a PARP-1 inhibitor (olaparib) could evoke enhanced apoptosis in HNSCC cells and/or result in sensitization to the DNA-damaging drug—cisplatin. 

## 2. Materials and Methods

### 2.1. Cell Lines and Culture Conditions

Commercially available HNSCC cells were used in the experiments: SCC-040 cells derived from tongue squamous cell carcinoma (German Collection of Microorganisms and Cell Cultures, DSMZ, Braunschweig, Germany), and FaDu cells derived from hypopharyngeal squamous cell carcinoma (American Type Culture Collection, ATCC, Manassas, VA, USA). The cells were cultured in high-glucose DMEM medium (Biowest, Nuaillé, France), supplemented with 5% FBS (EURx, Gdańsk, Poland), and 1% antibiotic solution (penicillin and streptomycin; Biowest, Nuaillé, France). The cells were maintained under standard conditions: 37 °C, 5% CO_2_, and 90% humidity in a Memmert incubator (Schwabach, Germany). 

### 2.2. Chemicals

The study employed four KDM inhibitors (KDMi): ML324 (targeting KDM4), CPI-455 (targeting KDM5), GSK-J4 (targeting KDM6), and JIB-04 (a pan-KDM inhibitor). Additionally, the platinum-based antineoplastic agent cisplatin and the PARP-1 inhibitor olaparib were utilized. All chemicals were purchased from Selleck Chemicals (Pittsburgh, PA, USA). Except for cisplatin, all compounds were dissolved in DMSO and stored in aliquots at −20 °C. Cisplatin stock solutions were prepared in saline and stored in light-protected tubes at room temperature for a maximum of 30 days. 

### 2.3. Cell Viability Assay

To assess the impact of the studied compounds and their combinations on cell viability, a resazurin assay was employed. Cells (6 × 10^3^ per well) were seeded onto black 96-well plates. The following day, fresh medium containing varying concentrations of compounds or their combinations was added. The control cells were treated with the vehicle. After 72 h of incubation, the medium was removed, and cells were washed with warm PBS buffer. Subsequently, a resazurin (Sigma-Aldrich, St. Louis, MO, USA) solution (1 μg/mL) was added to the culture medium. Fluorescence was measured after 90 min of incubation (excitation = 530 nm, emission = 590 nm) using the Infinite M200 multiplate reader (Tecan, Grödig, Austria). Each experiment was conducted independently at least three times, with nine separate replicates each time. 

### 2.4. Analysis of Apoptosis

The induction of apoptosis by the investigated chemicals and their combinations was assessed by flow cytometry with the Muse^®^ Annexin V and Dead Cell Kit (Merck, Darmstadt, Germany). This method relies on the externalization of phosphatidylserine as an apoptosis marker. The kit includes Annexin V for phosphatidylserine staining and 7-aminoactinomycin D (7-AAD) for counterstaining, facilitating the differentiation between early and late apoptotic cells. All procedures followed the manufacturer’s protocol. Briefly, 2 × 10^4^ cells were seeded into 24-well plates. After 24 h of pre-incubation, the growth medium was replaced with fresh medium containing the tested chemicals, or DMSO for the control sample. Following 48 h of incubation, cells were harvested, centrifuged, resuspended in culture medium, and stained with Annexin V and 7-AAD. After a 20 min incubation, samples were analyzed using the Muse^®^ Cell Analyzer (Merck, Darmstadt, Germany). 

### 2.5. Isolation of RNA and Gene Expression Analysis 

Total RNA was extracted from cells following 24 h of incubation with the investigated chemicals using the phenol-based reagent RNA Extracol (EURx, Gdańsk, Poland). Subsequently, the samples underwent reverse transcription using the smART First Strand cDNA Synthesis Kit (EURx, Gdańsk, Poland), following the manufacturer’s instructions. The impact of the compounds and their combinations on gene expression alterations was evaluated using the real-time PCR method, employing the SG qPCR Master Mix (EURx, Gdańsk, Poland) and the LightCycler 96 thermocycler (Roche, Basel, Switzerland). The PCR protocol included an initial enzyme activation step at 95 °C for 10 min, followed by 40 cycles comprising DNA denaturation at 95 °C for 15 s, primer annealing at 56 °C for 30 s, and elongation at 72 °C for 30 s with fluorescence measurement. The specificity of the reactions was confirmed through melting curve analysis. Transcript expression levels of genes associated with apoptosis and cell cycle regulation, including *BAX*, *CDKN1A*, *BIRC5*, *CCND1*, and *CCNA1*, were quantified using primers described previously [11,19]. The *TBP* and *PBGD* genes served as reference genes for relative expression calculations using the Pfaffl method. Samples from three independent experiments were analyzed in three replicates. The primer sequences used in the study are listed in Table 1.

### 2.6. DNA Damage Level Analysis

The potential of the investigated chemicals and their combinations to induce DNA damage was evaluated through flow cytometry analysis using the Muse^®^ H2A.X Activation Dual Detection Kit (Merck, Darmstadt, Germany). This method utilizes the phosphorylation of H2A.X histone at serine 139 as an indicator of DNA damage. The kit includes two conjugated antibodies (anti-histone H2A.X-PECy5 for measuring total H2A.X histone content, and Anti-phospho-histone H2A.X (Ser139)-Alexa FluorTM 555 targeting the activated form of H2A.X) which allow for simultaneous measurement of both total and phosphorylated protein levels. All procedures were conducted according to the manufacturer’s protocol. Briefly, 10^4^ cells were seeded into 12-well plates. Following 24 h of pre-incubation, the culture medium was replaced with a medium containing the investigated chemicals. After an additional 24 h of incubation, cells were collected and subjected to fixation and permeabilization. Subsequently, cells were treated with the antibody cocktail and incubated in the dark for 30 min. The cells were then resuspended in assay buffer and analyzed using the Muse^®^ Cell Analyzer (Merck, Darmstadt, Germany). Three independent experiments were performed.

### 2.7. Statistical Analysis

The Student’s *t*-test was utilized to determine whether differences between the experimental and control groups were significant. The one-way ANOVA test, followed by the Tukey post hoc test, was employed to analyze the differences between individual chemicals and their combinations. The changes were considered significant at a *p* value ≤ 0.05. 

## 3. Results

### 3.1. The Effects of the Compounds and Their Combinations on Cell Viability

**Figure 1:**
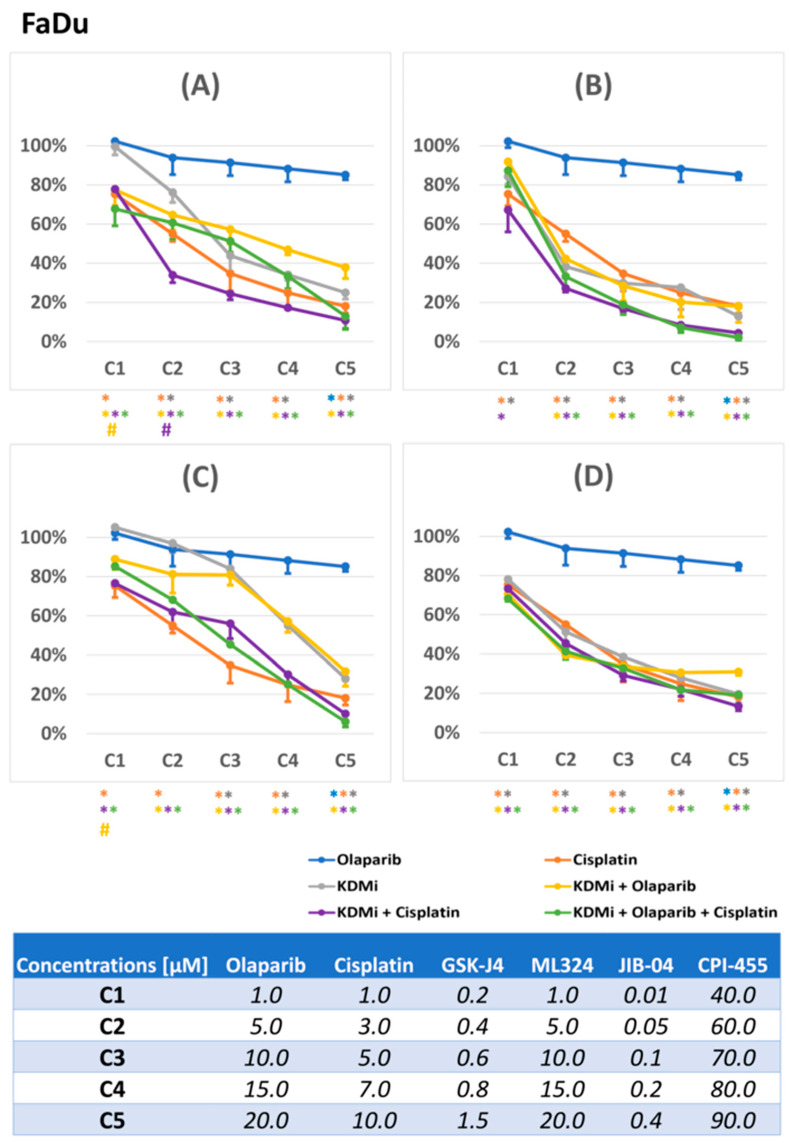
The effects of the investigated compounds and their combinations on the viability of FaDu cells measured by the resazurin assay. The KDMi ((**A**)—GSK-J4, (**B**)—ML324, (**C**)—JIB-04, (**D**)—CPI-455) were combined with olaparib, cisplatin, and both olaparib and cisplatin concurrently. Cells were treated with increasing concentrations of compounds for 72 h. Three independent experiments, with nine replicates per experiment, were performed. Charts (**A**–**D**) depict changes in relative viability ± SD (one-side error bar was used for transparency) compared to the control (cells treated with vehicle-DMSO). Symbols (*, #) in a given color refer to the compounds or combinations depicted in the legend using the same color—an asterix (*) denotes a significant difference in comparison to the DMSO control (*p* ≤ 0.05), while a hash (#) denotes that a given combination was significantly better at decreasing viability than each of its individual constituents alone (*p* ≤ 0.05). The table at the bottom of the figure presents the concentration of compounds used both as single agents and in combinations.

**Figure 2:**
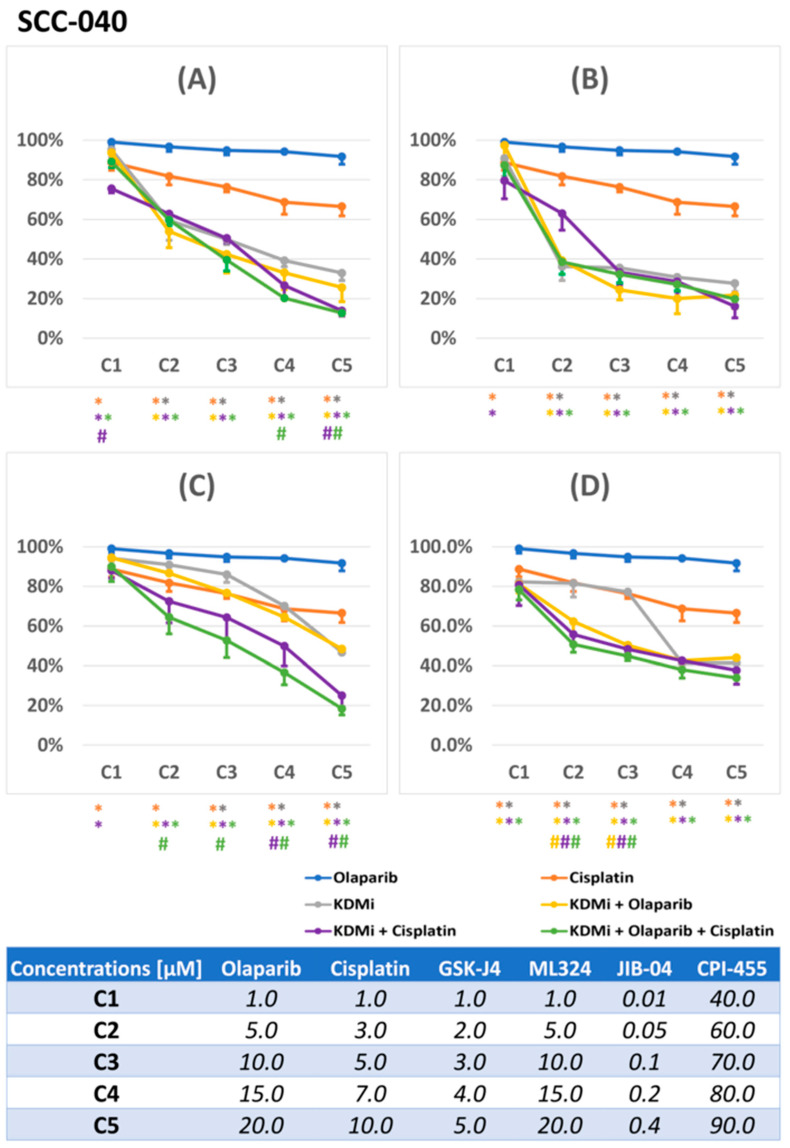
The effects of the investigated compounds and their combinations on the viability of SCC-040 cells measured by the resazurin assay. The KDMi ((**A**)—GSK-J4, (**B**)—ML324, (**C**)—JIB-04, (**D**)—CPI-455) were combined with olaparib, cisplatin, and both olaparib and cisplatin concurrently. Cells were treated with increasing concentrations of compounds for 72 h. Three independent experiments, with nine replicates per experiment, were performed. Charts (**A**–**D**) depict changes in relative viability ± SD (one-side error bar was used for transparency) compared to the control (cells treated with vehicle-DMSO). Symbols (*, #) in a given color refer to the compounds or combinations depicted in the legend using the same color—an asterix (*) denotes a significant difference in comparison to the DMSO control (*p* ≤ 0.05), while a hash (#) denotes that a given combination was significantly better at decreasing viability than each of its individual constituents alone (*p* ≤ 0.05). The table at the bottom of the figure presents the concentration of compounds used both as single agents and in combinations.

In the first step, we conducted viability assays to determine effective concentrations of the investigated compounds and observe the general effects of combining KDM inhibitors with olaparib and cisplatin in FaDu (Figure 1) and SCC-040 (Figure 2) cell lines. Olaparib, an approved orally administered drug with known pharmacokinetic parameters, was used at maximum concentrations that do not substantially exceed its typical plasma levels following standard administration [20]. At these concentrations, olaparib in monotreatment had little (and statistically insignificant) effect on FaDu and SCC-040 cell viability. In turn, cisplatin reduced the viability of FaDu cells to a greater extent.

We aimed to use equal concentrations of the compounds in both cell lines; however, due to pronounced differences in sensitivity to GSK-J4, we individualized GSK-J4 concentrations for each cell line. Generally, the SCC-040 cell line was more resistant to single-agent treatments, while combinatorial treatment seemed to exert more beneficial effects than in FaDu cells. Among single KDMi, JIB-04 demonstrated the greatest potency in both cell lines, exerting robust effects on cell viability in sub-micromolar concentrations (<0.5 µM), while CPI-455 was active in concentrations that might be challenging to reach in vivo (>50 µM).

Combining KDMi with olaparib had only a partly synergistic effect on cell viability in the studied cell lines. The viability curves for the double combinations of KDMi with olaparib were similar to those for KDMi alone; however, in FaDu cells, low concentrations of those compounds were more active than GSK-J4 and JIB-04 alone. Moreover, the double combinations of KDMi with cisplatin produced more promising results. In FaDu cells, we observed favorable outcomes for combinations of cisplatin with GSK-J4 and ML324. In SCC-040 cells, better results were seen with combinations of cisplatin with JIB-04 and CPI-455. The benefits of combining KDMi with both cisplatin and olaparib were most apparent in the SCC-040 cell line, where all triple combinations, except those containing ML324, demonstrated the greatest effectiveness.

The choice of the concentrations of compounds that were used in subsequent analyses was supported by our previous studies [11], and also by the results of the current viability assay, so as to warrant noticeable, but not too potent, effects, to allow discerning the potentiation of the effects in combinations of chemicals.

### 3.2. The Effects of the Compounds and Their Combinations on Apoptosis

**Figure 3:**
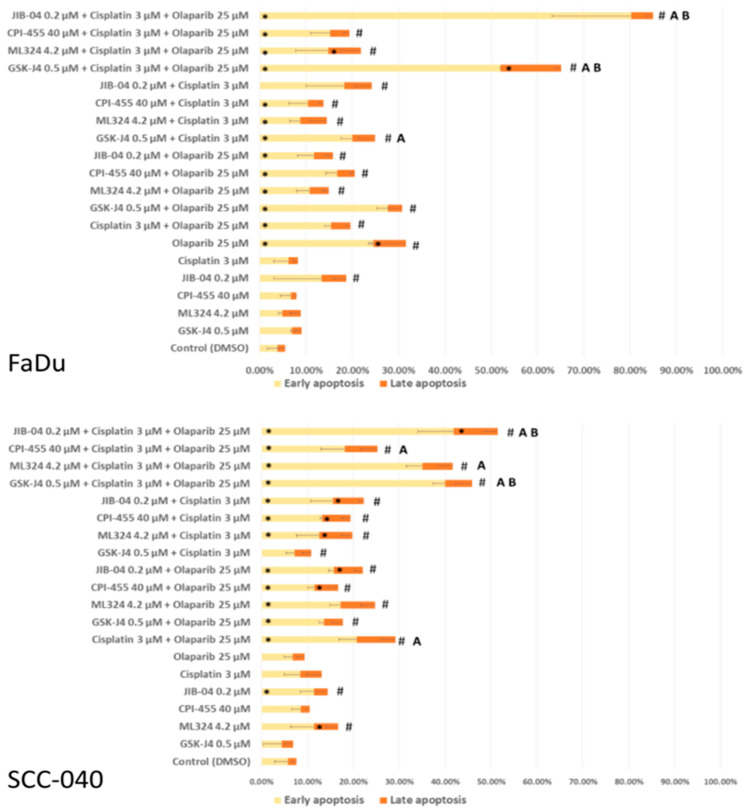
The results of the effect on apoptosis measured by Annexin V staining in FaDu and SCC-040 cell lines. The mean values ± SD from three independent experiments are shown. An asterisk (*) denotes statistically significant changes in the percentage of early or late apoptotic cells in comparison to the DMSO control, while a hash (#) denotes statistically significant changes in the total percentage of apoptotic cells in comparison to the DMSO control. Letter A denotes that a given combination was significantly better at inducing total apoptosis than each of its individual constituents alone. Letter B denotes that a triple combination of the given KDMi with cisplatin and olaparib was significantly better at inducing total apoptosis than all of the double combinations of this KDMi and the double combination of cisplatin and olaparib (*p* ≤ 0.05).

In the next step, we evaluated the pro-apoptotic effects of single compounds and their combinations with cisplatin and olaparib (Figure 3). In the FaDu cell line, single compounds, with the exception of olaparib, did not exert a statistically significant effect on the apoptosis levels in comparison to the control. However, all double and triple combinations, with the exception of the double combination of JIB-04 with cisplatin, exerted a significant induction of total apoptosis levels. 

Among the double combinations, simultaneous treatment of FaDu cells with GSK-J4 and cisplatin was the only one that exerted a greater effect on apoptosis induction than each of these compounds alone. We observed the greatest effect on the induction of apoptosis for triple combinations, especially for combinations containing GSK-J4 and JIB-04. For these combinations, the induction of apoptosis was significantly greater than for each of the compounds alone, but also significantly greater in comparison to double combinations with olaparib or cisplatin and double combinations of olaparib with cisplatin. 

In the SCC-040 cell line, general tendencies were similar; however, this cell line was generally more resistant to the investigated combinations. Induction of apoptosis after treatment with single compounds was not significantly different from control, with the exception of JIB-04 and ML324. Regarding the double combinations, all of them significantly induced total apoptosis compared to the control. However, only the combination of cisplatin with olaparib led to a greater induction of apoptosis than each of the compounds alone. Similar to the effects observed in the FaDu cell line, the most effective of the triple combinations were those containing GSK-J4 and JIB-04. Additionally, as in the FaDu cell line, these combinations were significantly better at inducing apoptosis compared to individual compounds and double combinations.

### 3.3. The Effects of the Compounds and Their Combinations on DNA Damage Level

**Figure 4:**
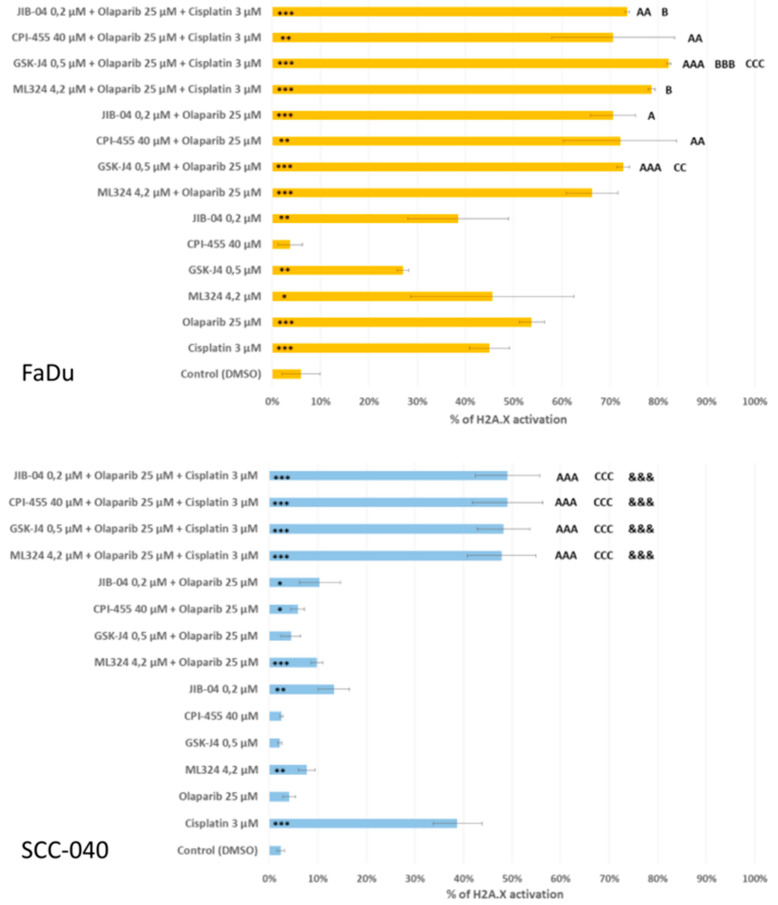
The mean values ± standard deviation (SD) from three independent experiments are presented. An asterisk (*) indicates statistically significant changes in H2A.X activation percentages compared to the DMSO control. Additionally, letters (A, B, C) denote significant differences between various combinations and individual agents, such as the KDM inhibitor (A), cisplatin (B), or olaparib (C). Statistically significant differences between double and triple combinations are denoted by the ampersand symbol (&). Significance levels are represented by one (*p* ≤ 0.05), two (*p* ≤ 0.01), or three (*p* ≤ 0.001) letters (A, B, C) or symbols (*, &).

Our subsequent analysis aimed to evaluate the potential of the investigated compounds and combinations to induce DNA damage, using the activation of H2A.X as the marker (Figure 4). Interestingly, all individual compounds, except CPI-455, were able to induce DNA damage in FaDu cells. Double combinations of KDMi with olaparib generally presented a higher rate of H2A.X activation than single compounds, although only the combination of GSK-J4 with olaparib produced a significant increase in comparison to both of the compounds alone. In the FaDu cell line, none of the triple combinations was significantly more effective at inducing DNA damage than the corresponding double combinations. In the SCC-040 cell line, individual compounds at the studied concentrations led to significantly increased DNA damage levels only in the cases of cisplatin, ML324 and JIB-04. Double combinations of KDMi with olaparib did not produce significantly different effects from individual compounds. While triple combinations exerted significantly greater effects than KDMi, olaparib, and corresponding double combinations, none of them led to a significantly greater accumulation of DNA damage than monotreatment of SCC-040 cells with cisplatin.

### 3.4. The Effects of the Compounds and Their Combinations on Gene Expression Level

**Figure 5:**
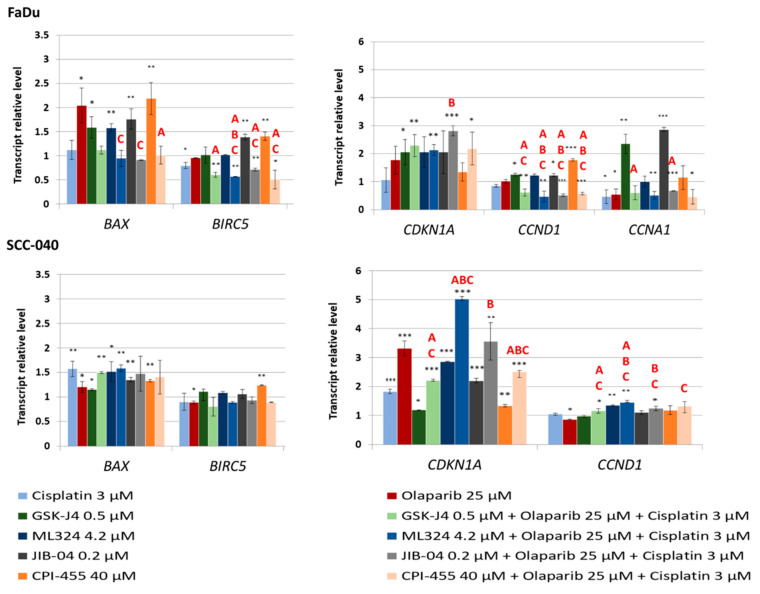
The effect of the investigated KDMi and their triple combinations with cisplatin and olaparib on the relative transcript level of genes associated with apoptosis and cell cycle regulation. The mean values ± SD from three independent experiments with three replicates per experiment are presented. The asterisk above the bar denotes statistically significant changes in comparison to the DMSO control (* *p* ≤ 0.05, ** *p* ≤ 0.01, *** *p* ≤ 0.001). Letters A, B, and C denote statistically significant changes in comparison to KDMi (A), cisplatin (B), and olaparib (C) alone (*p* ≤ 0.05).

Since our previous analysis found that the combinations of KDMi with cisplatin and olaparib are superior at inducing apoptosis, we decided to evaluate the effects of KDMi and their triple combinations with cisplatin and olaparib on the expression of genes related to apoptosis and cell cycle regulation (Figure 5). 

In the FaDu cell line, cisplatin alone decreased the expression of *BIRC5*, without affecting *BAX* expression. Conversely, olaparib alone increased *BAX* expression without altering *BIRC5* transcript levels. Interestingly, in FaDu cells, all the investigated KDMi alone increased *BAX* expression, whereas in combinations, *BAX* transcript levels remained similar to those in the control. KDMi alone were not effective at reducing *BIRC5* expression in FaDu cells, while all triple combinations significantly downregulated *BIRC5* expression. In SCC-040 cells, individual compounds and their combinations slightly induced *BAX* expression, but none significantly decreased *BIRC5* expression.

Triple combinations generally promoted *CDKN1A* expression more effectively than individual compounds in both cell lines. However, these differences were significantly greater than for individual compounds of the combination only in SCC-040 cells for the combinations containing ML324 and CPI-455. In FaDu cells, individual KDMi slightly induced *CCND1* expression, while triple combinations significantly decreased *CCND1* transcript levels. In SCC-040 cells, none of the KDMi alone or in combination led to the downregulation of *CCND1*. Interestingly, in FaDu cells, GSK-J4 and JIB-04 alone led to a pronounced induction of *CCNA1* expression, while triple combinations reduced *CCNA1* transcript levels, exerting similar effects to cisplatin and olaparib alone. In SCC-040 cells, *CCNA1* expression was not detected, even in control cells.

## 4. Discussion

There are ongoing efforts to improve the efficacy of treatment for patients with locoregionally advanced HNSCC, who frequently require the addition of chemotherapy to standard surgery and radiotherapy regimens [21]. Based on the observation that PARP inhibition exerts synthetic lethality in HR-deficient cancer cells, it was suggested that PARP inhibitors could be used as cisplatin chemosensitizers in a subset of HNSCC patients who present HR-deficiency [6]. However, the majority of HNSCC tumors are HR-proficient [9] and thus not easily treatable with PARP inhibitors. Interestingly, recent findings showed that HR deficiency can be induced pharmacologically, and KDM inhibitors constitute promising modulators of DNA damage response, including HR repair [14,22]. Therefore, we evaluated two hypotheses in our study. The first hypothesis was prompted by the reports that specific KDMi can induce DNA damage in cancer cells and disrupt DNA repair mechanisms, including homologous recombination [16,23]. We hypothesized that due to the ability of KDMi to disrupt homologous recombination, their combination with the PARP inhibitor olaparib would result in a synergistic effect akin to synthetic lethality, as seen in the treatment of *BRCA*-mutated cancers. Our second hypothesis was based on the assumption that simultaneous pharmacological disruption of multiple DNA repair mechanisms by the joint use of KDMi and olaparib could increase the susceptibility of HNSCC cancer cells to the DNA-damaging drug cisplatin. 

The results of the conducted analyses allowed us to refute the first hypothesis, as both the viability assay and the apoptosis assay did not reveal significant potentiation of the effects between olaparib and KDMi. This is in contrast to a study that reported that another KDM inhibitor, VLX600, showed synergism with olaparib; however, it was performed using HR-proficient ovarian cancer cells [22]. On the other hand, we found that three out of four of the investigated KDMi (GSK-J4, ML324, and JIB-04) indeed increased DNA damage levels in the investigated cell lines, when they were individually applied. We assessed DNA damage by evaluating the level of H2A.X phosphorylation, which was suggested as a marker of response to PARP inhibitors [10]. Indeed, our results showed stronger induction of apoptosis by olaparib in FaDu cells, which was accompanied by a higher elevation in the γH2A.X level. On the other hand, the increase in the level of phosphorylation of H2A.X did not reflect the effect on apoptosis induction in the case of cisplatin, suggesting that this marker cannot be indiscriminately regarded as a measure of drug response to these diverse chemicals. The inhibitors of KDM also affected DNA damage in a cell-line dependent manner. These results are consistent with other studies in different cancer models. The induction of DNA damage by individually applied GSK-J4 is in line with recent findings that the lack of KDM6 function affects DNA damage response [24]. Moreover, it was demonstrated that GSK-J4 can impair DNA damage repair mechanisms in glioma cells [23], and that JIB-04 can lead to DNA damage accumulation in lung cancer and Ewing sarcoma models [16,25]. Interestingly, JIB-04 was much more potent than GSK-J4 in attenuating the resolution of radiation-induced γH2A.X foci in lung cancer cells [26]. While there is evidence showing that KDM4B is recruited in DNA repair processes [27], to our knowledge, our study is the first to demonstrate that inhibition of KDM4 with ML324 can lead to the accumulation of DNA damage in cancer cells.

On the other hand, the results of this study support our second hypothesis, that combining KDMi with cisplatin and concurrently with both cisplatin and olaparib can lead to an enhanced cytotoxic effect in cancer cells. Combining cisplatin with PARP inhibitors is also a well-investigated strategy, yielding promising results, in head and neck cancer models [28,29,30,31,32]. In addition, there is some data on the beneficial effects of combining KDMi with cisplatin, although not in head and neck cancers [16,33,34]. However, to our knowledge, triple combinations of KDMi with PARP inhibitors and cisplatin were not investigated in any experimental settings. In our study, all the investigated KDMi decreased viability and induced apoptosis in the examined cell lines, with JIB-04 demonstrating the greatest potency. Importantly, combining KDMi with cisplatin enhanced the cytotoxic effect. On the other hand, triple combinations of KDM inhibitors (especially GSK-J4 and JIB-04) with cisplatin and olaparib exhibited the greatest cytotoxic activity in our experiments. The induction of apoptosis by the triple combinations of KDMi with olaparib and cisplatin was not straightforwardly related to the impairment of the HR repair pathway, which was indirectly evaluated by γH2A.X level measurement in our study. This possibly indicates that factors other than DNA damage response modulation are also implicated in the observed proapoptotic effects. Interestingly, we found that potentiation of anti-tumor effects by combining KDMi with cisplatin and olaparib had different mechanisms at the level of gene expression in the investigated cell lines. In FaDu cells, it was related to decreasing the expression of the *BIRC5* gene, which encodes the pro-survival protein survivin, and to downregulating the expression of the cyclins, particularly *CCND1*. On the other hand, in the SCC-040 cell line, potentiation of effects observed for triple combinations was related to the induction of *CDKN1A* expression, which encodes the p21 protein, related to cell cycle arrest. In general, the joint inhibition of KDM4–6 by the pan-KDM inhibitor JIB-04 warranted the best results in drug combinations, which was followed by the inhibition of KDM6 by GSK-J4, which may suggest that all three classes of KDMs are crucial in modulating DNA damage response and cisplatin sensitivity in head and neck cancer cells, and may point to the importance of KDM6. Thus, with respect to previous suggestions that PARP inhibitors could be used as cisplatin chemosensitizers in HNSCC patients [6], the results of this study imply that the addition of KDM4–6 inhibitors could further boost the chemosensitizing effects. This has to be further evaluated using additional experimental models, including ex vivo organoid cultures or in vivo animal studies.

Unavoidably, this research entails some limitations. In all assays, besides viability analysis, the activity of compounds alone and in combinations was assessed for single concentrations, while synergistic effects can vary across different concentration ranges. Additionally, we evaluated only one treatment mode, which is the concurrent treatment of cancer cells with all the compounds of a particular combination, which entails the risk of chemical interaction between the compounds. Thus, future studies could explore other treatment modes, and a more diverse set of concentrations of the studied chemicals. Moreover, to fully understand the potential of this therapeutic approach, it would also be interesting to investigate the mechanisms of inducing DNA damage by KDMi and the investigated combinations by performing analyses that assess the mechanisms of DNA damage induction and the efficiency of different DNA repair pathways. Future research could address these issues. However, these limitations do not affect the main observations of this study.

## 5. Conclusions

In conclusion, the combinations of the PARP inhibitor olaparib with the tested KDM inhibitors did not exhibit the expected efficacy against HNSCC cells and cannot be developed into an effective strategy for HNSCC treatment. However, double combinations of KDM inhibitors (particularly JIB-04 and GSK-J4) with cisplatin, and triple combinations with cisplatin and olaparib, showed potentiation of cytotoxic and proapoptotic effects, representing promising therapeutic strategies for HNSCC treatment. Thus, the chemo-sensitizing potential of the inhibitors of KDMs 4–6, and their combinations with olaparib, should be further evaluated in HNSCC cells. In particular, the triple combinations of JIB-04 or GSK-J4 with olaparib and cisplatin seem to hold the greatest potential for a clinical benefit in HNSCC patients, which requires further exploration and in vivo confirmation.

## Figures and Tables

**Table 1:** Sequences of primers used in RT-qPCR.

Gene	Forward Primer (5′ to 3′)	Reverse Primer (5′ to 3′)
*BAX*	GCTTCAGGGTTTCATCCAG	GGCGGCAATCATCCTCTG
*CDKN1A*	CAGACCAGCATGACAGATTT	TTCCTGTGGGCGGATTAG
*BIRC5*	GGACCACCGCATCTCTAC	CCTTGAAGCAGAAGAAACAC
*CCND1*	CCCTCGGTGTCCTACTTC	TCCTCGCACTTCTGTTCC
*CCNA1*	TTCATGTATGTCTGTTCTG	GTCTACTTCAGGAGGATAT
*PBGD*	TCAGATAGCATACAAGAGACC	TGGAATGTTACGAGCAGTG
*TBP*	GGCACCACTCCACTGTATC	GGGATTATATTCGGCGTTTCG

## Data Availability

The original contributions presented in the study are included in the article, further inquiries can be directed to the corresponding author.
